# Identification of novel miRNAs and miRNA expression profiling in embryogenic tissues of *Picea balfouriana* treated by 6-benzylaminopurine

**DOI:** 10.1371/journal.pone.0176112

**Published:** 2017-05-09

**Authors:** Qingfen Li, Cheng Deng, Yan Xia, Lisheng Kong, Hanguo Zhang, Shougong Zhang, Junhui Wang

**Affiliations:** 1 Guangdong Key Laboratory for Innovative Development and Utilization of Forest Plant Germplasm, State Key Laboratory for Conservation and Utilization of Subtropical Agro-bioresources, College of Forestry and Landscape Architecture, South China Agricultural University, Guangzhou, China; 2 State Key Laboratory of Tree Genetics and Breeding, Key Laboratory of Tree Breeding and Cultivation of State Forestry Administration, Research Institute of Forestry, Chinese Academy of Forestry, Beijing, China; 3 Department of Biology, Centre for Forest Biology, University of Victoria, Victoria, British Columbia, Canada; 4 State Key Laboratory of Tree Genetics and Breeding, Northeast Forestry University, Harbin, China; Kunming University of Science and Technology, CHINA

## Abstract

Here, we compared miRNA expression profiles in embryonic cell cultures of the conifer *Picea balfouriana* following application of the synthetic cytokinin 6-benzylaminopurine (6-BAP). We used next-generation sequencing to analyze three libraries of small RNAs from the treated embryogenic cell cultures and generated 24,000,000 raw reads from each of the libraries. Over 70 differentially regulated micro RNA (miRNA) families (≥2 fold change in expression) were identified between pairs of treatments. A quantitative analysis showed that miR3633 and miR1026 were upregulated in tissues with the highest embryogenic ability. These two miRNAs were predicted to target genes encoding receptor-like protein kinase and GAMYB transcription factors, respectively. In one library, miR1160, miR5638, miR1315, and miR5225 were downregulated. These four miRNAs were predicted to target genes encoding APETALA2, calmodulin-binding protein, and calcium-dependent protein kinase transcription factors. The expression patterns of the miRNAs and their targets were negatively correlated. Approximately 181 potentially novel *P*. *balfouriana* miRNAs were predicted from the three libraries, and seven were validated during the quantitative analysis. This study is the first report of differential miRNA regulation in tissues treated with 6-BAP during somatic embryogenesis. The differentially expressed miRNAs will be of value for investigating the mechanisms of embryogenic processes that are responsive to 6-BAP in *P*. *balfouriana*.

## Introduction

Somatic embryogenesis (SE) is an important method in spruce breeding programs and enables the regeneration and cloning of trees with desirable genotypes. However, the callus tissue formed from some genotypes can gradually lose embryogenic capacity. The mechanism of this effect is unclear, as the early stages of SE are insufficiently known [1–3]. This mechanism has been investigated by identifying differentially expressed genes between embryogenic and nonembryogenic tissues in order to elucidate the molecular regulatory networks that operate during SE in plant species [4], including *Picea abies* (L.) H. Karst. [5], *Picea glauca* (Moench) Voss [6], *Glycine max* (L.) Merr. [7], *Gossypium hirsutum* L. [8], *Zea mays* L. [9], *Solanum tuberosum* L. [10], *Triticum aestivum* L. [11], and *Citrus* × *sinensis* (L.) Osbeck [12]. However, the genetic regulation of these differentially expressed genes and their specific functions remain largely unknown.

Microribonucleic acids (miRNAs) are a group of endogenous small RNA (sRNA) molecules, generally 20–25 nucleotides (nt) in length, that play essential roles in most eukaryotes by regulating the expression of their target genes [13]. MiRNAs extensively affect biological processes in plants, primarily development and stress responses [14]. Notably, SE is also regulated by miRNAs and their roles have been studied [15–22] including in gymnosperms [23–24]. Wu et al. [19] found that the target genes of the miRNAs miR164, miR166, and miR397 were associated with the formation of nonembryogenic callus. Moreover, the effects of miR166a overexpression on the development of SEs in *Larix leptolepis* has been investigated [25]. Su et al. [26] reported that overexpression of miR167 inhibited SE formation, showing that miR167 negatively regulates SE induction. Zhang et al. [27] reported that miR165 was differentially expressed between embryogenic and nonembryogenic callus. Wu et al. [28] found that the conserved miRNAs csi-miR156a/b, miR164b, and 171c directly suppressed expression of a specific transcription factor, and were suggested to inactivate postembryonic growth to maintain normal SE. Zhang et al. [29] showed that miR171a/b may influence the function of proembryogenic masses (PEMs), while miR171c may have a role in SE induction in larch; miR397 and miR398 were found to be primarily involved in modulation of PEM propagation and transition to single embryos. The transcription factor gene *SQUAMOSA Promoter-Binding Protein-Like* is regulated by miR156; this transcription factor acts as a pleiotropic regulator of plant development and can promote vegetative phase transition by activating miR172 [30]. Li et al. [31] concluded that in *Larix kaempferi* (Lamb.) Carrière, the post-transcriptional regulation of *MYB33* by miR159 was associated with the maintenance of embryogenic or nonembryogenic potential and somatic embryo maturation.

*Picea balfouriana* Rehder & E.H. Wilson grows in many parts of the world and is prevalent in southwestern and eastern regions of China. It is a species of spruce that has ecological and economic importance, and produces high-quality wood. To date, transcriptomic and proteomic approaches have been applied to unravel the molecular mechanisms of SE in *P*. *balfouriana* [1, 2]. We showed that 6-BAP, a synthetic cytokinin, had a significant influence on embryogenic competence during the proliferation stage [2]. Our earlier analysis indicated that 6-BAP has a significant effect on proteins and mRNAs. However, the mRNAs of 15% of proteins have not been identified in *P*. *balfouriana* mRNA libraries. In the present study we sought to obtain a better understanding of the effect of 6-BAP on the molecular regulation of SE as this could be of value for spruce seedling production. To this end, we compared miRNA expression patterns among embryogenic cultures that had received different 6-BAP treatments.

## Materials and methods

### Plant materials

A *P*. *balfouriana* embryogenic cell line was initiated in 2011 using seeds from elite genotype 4 at the Research Institute of Forest, Chinese Academy of Forestry (Beijing, China). Cells from this line were placed in half-strength LM medium as an induction medium [32], which was supplemented with 10 μM 2,4-d-ichlorophenoxyacetic acid and 5.0 μM 6-BAP [33], 500 mg·L^−1^ glutamine, 1g·L^−1^ casein hydrolysate, 1% sucrose, and 8% agar, at 24 ± 1°C in the dark. The proliferation medium was half-strength LM medium with three concentrations of 6-BAP (2.5 μM, 3.6 μM, and 5.0 μM); the other supplements and culture conditions were the same as for the induction medium. After three months, embryogenic tissues with different embryogenic capabilities were produced. The embryogenic cultures were subcultured at two-week intervals. The SE culture experiment was performed twice. The embryo differentiation method has been described previously [3].

Samples of the embryogenic cultures were collected after being subcultured for 7 d. For each treatment, three and six biological replicates for physiological and sRNA profiling, respectively, were collected. All samples were transferred to cryotubes, flash frozen in liquid nitrogen (N_2_), and stored at -80°C until further processing for metabolite extraction.

### Plant hormone determination

The extraction, purification, and determination of endogenous levels of indole-3-acetic acid (IAA), zeatin riboside (ZR), gibberellic acid (GA_3_), and ABA were performed by an indirect enzyme-linked immunosorbent assay (ELISA) technique as described previously [34]. Briefly, a 0.5 g sample of each treatment was homogenized in liquid N_2_ and extracted in cold 80% (v/v) methanol with butylated hydroxytoluene (1 mmol·L^−1^) overnight at 4°C. The supernatant was collected after centrifugation at 1,500 × *g* (4°C) for 8 min, passed through a C_18_Sep-Pak cartridge (Waters, Milford, MA), and dried under N_2_. The residue was dissolved in phosphate buffered saline (0.01 mol·L^−1^, pH 7.4) and the levels of IAA, ZR, GA_3_, and ABA were determined. Microtitration plates (Nunc, Denmark) were coated with synthetic IAA, ZR, GA_3_, or ABA ovalbumin conjugates in NaHCO_3_ buffer (50 mmol·L^−1^, pH 9.6) and left overnight at 37°C. Then, ovalbumin solution (10 mg·mL^−1^) was added to each well to block nonspecific binding. After incubation for 30 min at 37°C, standard IAA, GA_3_, ABA, and ZR samples and antibodies were added and incubated for a further 30 min at 37°C. The antibodies against IAA, ZR, GA_3_, and ABA were obtained as described by Weiler et al. [35]. In addition, horseradish peroxidase-labelled goat anti-rabbit immunoglobulin was added to each well and incubated for 1 h at 37°C. Finally, the buffered enzyme substrate (ortho-phenylenediamine) was added, and the enzyme reaction was carried out in the dark at 37°C for 15 min, then terminated using 3 mol·L^−1^ H_2_SO_4_. Absorbance was recorded at 490 nm. Analyses of the enzyme-immunoassay data followed the procedures described in Weiler et al. [35].

### RNA isolation and purification

Total RNA was isolated and purified from tissues at the proliferation stage of SE using a Total RNA Purification kit (Norgen Biotek Corporation, Canada) according to the manufacturer’s instructions, following the on-column DNA removal protocol. All RNA samples from tissues treated with three concentrations of 6-BAP were stored at -80°C until sRNA sequencing was performed and were mixed in equal ratios to form a single RNA pool.

### Sequencing and data analysis

Three sRNA libraries were generated from the different 6-BAP-treated tissues and were sequenced on a HiSeq 2000 Sequencing System (Illumina, San Diego, CA, USA) to identify conserved and novel miRNAs. First, sRNA fragments (16–30 nt) were isolated from a 15% polyacrylamide electrophoresis gel and purified. Then the sRNAs were sequentially ligated to a 5′RNA adapter (5′-GUUCAGAGUUC UACAGUCCGACGAUC-3′) and a 3′RNA adapter (5′-pUCGUAUGCCGUCUUCUGCUUGidT-3′: p, phosphate; idT, inverted deoxythymidine) using T4 RNA ligase. The resulting adaptor-ligated sRNAs were reverse transcribed to cDNA with a reverse transcription primer (5′-CAAGCAGAAGAC GGCATACGA-3′) using Superscript II reverse transcriptase (Invitrogen) and amplified by polymerase chain reaction (PCR). The cDNA was sequenced on an Illumina/Solexa sequencing platform by the Beijing Genomics Institute (Shenzhen, China).

Bioinformatics tools were used to analyze the sequencing data. The 35-nt sequence tags were first trimmed of adaptors, regions of low complexity, and low-quality sequences; then, the length distribution of clean tags was summarized. The remaining sRNA sequences (clean reads) were mapped to the transcriptomes of *P*. *balfouriana* embryogenic cultures (PRJNA211928 and PRJNA248161). The unique sRNA sequences were searched against known miRNA sequences in miRBase (Release 21, http://www.mirbase.org/) to identify conserved miRNAs in *P*. *balfouriana*. Mireap software was used to predict novel miRNAs among the sRNAs that did not match any of the sequences in miRBase, and the secondary structures of the putative novel miRNAs were predicted using Mfold 3.1 [22]. At the same time, because there was bias (5′ U) in the first-position base of miRNA, we estimated prediction accuracy by calculating this first position base bias among sRNA candidates 18–24 nt in length. Additionally, the target genes of potential novel and conserved miRNAs were predicted as described previously [36–38] and their potential roles in early SE were investigated by functional annotation using the Gene Ontology (GO) and Kyoto Encyclopedia of Genes and Genomes (KEGG) Pathway databases.

### Selection of candidate reference miRNAs

Five miRNA genes, U6 snRNA, dlo-miR24, dlo-miR168a*, Csi-snoR14, and 5.8S ribosomal RNA (rRNA), were selected because they have been reported to be the most stable reference RNA genes for miRNA quantitative reverse-transcription (qRT)-PCR studies [20,24]. Three different gene normalization applets, geNorm [39], BestKeeper [40], and NormFinder [41], were used to analyze the expression stability of the candidate reference RNA genes. The geNorm software calculates a gene stability measure (M) for each gene in a given set of samples via stepwise exclusion of the least stable genes. The preliminary analysis of BestKeeper, based on the inspection of raw threshold cycle values, estimates the variation of all the reference genes using correlation analyses to develop a weighted index, and gives standard deviation values < 1. NormFinder is based on an analysis of variance mathematical model to estimate intra- and intergroup variation that calculates reference gene stability values. Based on the results from the three applets, we selected the most stable gene as the reference gene in this study.

### Real-time quantitative PCR of miRNAs and their targets

The expression levels estimated from the high-throughput sequencing data of *P*. *balfouriana* miRNAs were validated by qRT-PCR. RNA samples from the 6-BAP-treated embryogenic cultures were reverse transcribed using an NCode VILO miRNA cDNA Synthesis kit (Invitrogen, USA). A total of 20 miRNAs (including conserved and novel) were examined using an NCode Express SYBR Green ER miRNA qPCR kit (Invitrogen). All the reactions were performed in triplicate in a STEPONE PLUS Real Time PCR System (Applied Biosystems, USA), with a dissociation curve used to control for primer dimers in the reactions. Mature miRNA abundance was calculated relative to the expression of the selected reference gene. The selected miRNAs and primer sequences are listed in S1 Table.

Total RNA was extracted as described above, and qRT-PCR was carried out to assess the abundance of 30 of the miRNA target mRNAs. The mRNA qRT-PCR was performed using a RevertAid First Strand cDNA Synthesis Kit (Fermentas, Thermo Fisher, USA) and a SYBR Premix EX Taq Kit (TaKaRa Biotechnology, Japan), following the manufacturers’ instructions. All expression levels were normalized to the expression of the reference gene WS0109_C05 (peroxisomal targeting signal receptor). The 30 target mRNAs and their primer sequences are listed in S2 Table. Reactions were performed on an ABI Step-One Plus Real Time PCR System (Applied Biosystems) with three replicates for each sample. After PCR, dissociation curves and amplification curves were analyzed to verify the specificity of the amplification. The data were analyzed with Microsoft Excel2007 using Student’s t-test, and P-values of less than 0.05 were considered significant.

## Results

### Development of *P*. *balfouriana* somatic embryos

During the maturation stage, embryogenic tissue from the *P*. *balfouriana* cell line exhibited different embryogenic abilities after treatment with 2.5 μM, 3.6 μM, or 5.0 μM 6-BAP (Table 1). The highest rates of embryogenic tissue proliferation occurred in medium containing the intermediate concentration (3.6 μM) of 6-BAP, which yielded the most fully mature embryos with a normal set of cotyledons (113 ± 6 per 100 mg tissue). This culture also had the highest germination rate (48.47% ± 0.03) of the three treatments. Tissue in medium containing 5.0 μM 6-BAP produced the lowest number of somatic embryos (23 ± 2 per 100 mg tissue) and had the lowest germination rate (9.42% ± 0.02). Tissues treated with 2.5 μM or 3.6 μM 6-BAP had higher embryogenic competence than those treated with 5.0 μM 6-BAP.

**Table 1:** Number of mature cotyledonary embryos generated from each 6-BAP treatment and their germination rates.

6-BAP concentrations	Mature embryos per 100 mg of embryogenic tissue	Germination rate
**2.5 μM**	89 ± 3^b^	31.78% ± 0.02^b^
**3.6 μM**	113 ± 6^a^	48.47% ± 0.03^a^
**5.0 μM**	23 ± 2^c^	9.42% ± 0.02^c^

Superscript letters (a, b, and c) indicate significant differences (*p* ≤ 0.05).

**Fig 1:**
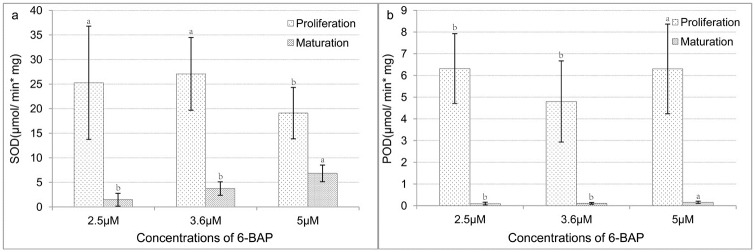
Activities of antioxidant enzymes in 6-BAP-treated tissues in proliferation and maturation stages. **(a)** Activity of superoxide dismutase (SOD). **(b)** Activity of peroxidase (POD). Tissues in proliferation and maturation stages were collected after being transferred to new media for 7 d. Lowercase letters (a, b, c) indicate significant differences (*p* ≤ 0.05).

In the proliferation stage, superoxide dismutase (SOD) activity in the 2.5 μM and 3.6 μM treatments was significantly higher than in the 5.0 μM treatment, whereas in the maturation stage, SOD activity was significantly lower with 2.5 μM and 3.6 μM than with 5.0 μM 6-BAP (Fig 1a). Peroxidase (POD) activity was contrary to that of SOD (Fig 1b). Interestingly, there were significant differences in SOD and POD activities between embryogenic tissues treated with different concentrations of 6-BAP in the proliferation and maturation stages.

**Fig 2:**
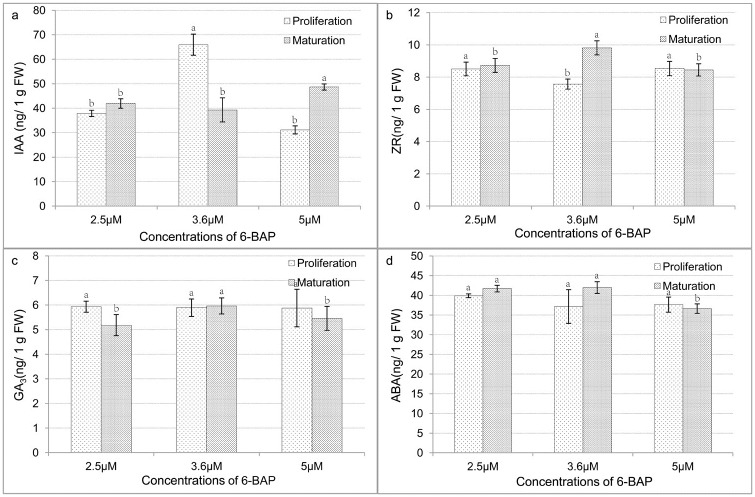
Levels of plant hormones in 6-BAP-treated tissues in proliferation and maturation stages. **(a)** Levels of 3-acetic acid (IAA). **(b)** Levels of zeatin-riboside (ZR). **(c)** Levels of gibberellic acid (GA_3_). **(d)** Levels of abscisic acid (ABA). Tissues in proliferation and maturation stages were collected after being transferred to new media for 7 d. Lowercase letters (a, b, c) indicate significant differences (*p* ≤ 0.05).

In the proliferation stage, IAA and ZR levels in tissues with the highest embryogenic ability (3.6 μM 6-BAP) were significantly higher and lower, respectively, than in the other two treatments (Fig 2). There were no significant differences in the contents of GA_3_ and ABA among the three treatments in the proliferation stage; however, there were significant differences in the maturation stage. The contents of GA_3_ and ABA in the 3.6 μM and 2.5 μM treatments were significantly higher than in the 5.0 μM treatment.

## High-throughput sequencing of sRNAs

**Fig 3:**
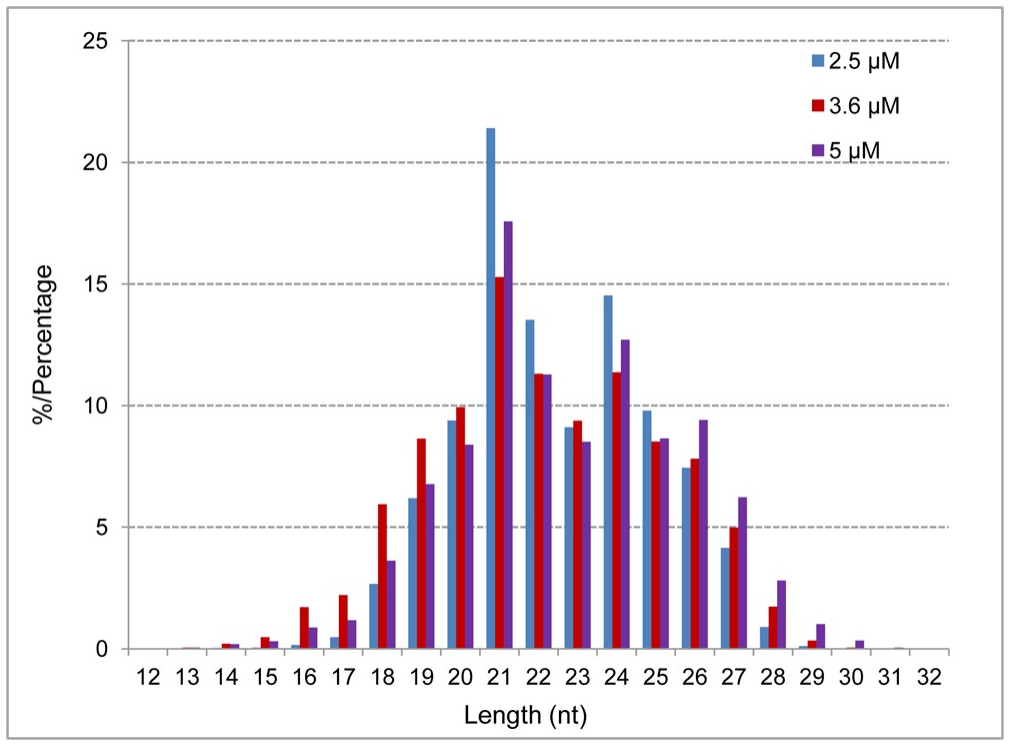
Size distribution analysis of the sRNA sequences in three *Picea balfouriana* libraries.

The Illumina/Solexa sequencing generated 25,518,179 reads from the 2.5 μM library, 25,588,136 reads from the 3.6 μM library, and 24,417,174 reads from the 5.0 μM library after removing the empty adapters and low-quality sequences (data are available via Sequence Read Archive [SRA] with identifier PRJNA248161, https://www.ncbi.nlm.nih.gov/sra/?term=PRJNA248161). Only 3.18%, 21.18%, and 4.23% of the unique sRNA sequences from the 2.5 μM, 3.6 μM, and 5.0 μM libraries, respectively, mapped to the *P*. *balfouriana* transcriptomes (Fig 3). The unique sRNAs were then compared against all plant precursor and mature miRNAs listed in miRBase and 27,362 (0.85%), 30,165 (1.05%), and 27,479 (1.02%) of the unique sequences from the 2.5 μM, 3.6 μM, and 5.0 μM libraries, respectively, were found to be similar to known miRNAs. A BLASTN search against the Rfam database identified rRNAs, small nuclear RNAs (snRNAs), small nucleolar RNAs (snoRNAs), and tRNAs among the unique sRNA sequences in our datasets. However, most unique sRNA sequences (96.82%, 78.82%, and 95.77% in the 2.5 μM, 3.6 μM, and 5.0 μM libraries, respectively) could not be annotated; this difficulty is consistent with results obtained in similar studies in other plants [24, 42–45]. The primary reason for the high percentage of unannotated sequences may be the limited number of species-level genomes and expressed sequence tags in these databases.

The length distributions of the sRNAs in the three libraries were similar (Fig 3). The unique sRNAs were 12–32 nt long with 21 nt predominating (21.4%, 15.28%, and 17.57%), followed by 24 nt (14.63%, 11.37%, and 12.70%), and 22 nt (13.52%, 11.31%, and 11.28%). This pattern of length distribution is consistent with those reported for tomato [46], wheat [47], *Populus* [48], and most other angiosperms [43,45,49]. The ratio of 21 to 24 nt lengths is highly variable among plants, indicating that significant differences exist in sRNA biogenesis pathways among different species [50].

## Conserved miRNAs in *P*. *balfouriana* callus

By comparing the sRNA sequences in the three libraries to known mature plant miRNAs in miRBase, we obtained 6,050 miRNAs (250 families), 4,984 miRNAs (214 families), and 5,381 miRNAs (229 families) in the 2.5 μM, 3.6 μM, and 5.0 μM libraries, respectively. Notably, more than 2,000 of the miRNAs matched in miRBase had no annotation in each library. Approximately 90% of the identified miRNAs were shared in the three libraries, and the same nine miRNA families (miR156, 166, 396, 951, 950, 946, 3712, 1312, and 1314) were shared among the first 10 most abundant miRNA families in each library.

## Prediction and classification of targets of differently regulated miRNAs in *P*. *balfouriana*

More than 4% of the miRNAs appeared in only one of the libraries and more than 70 differentially expressed miRNA families (≥ 2 fold change) were identified between any two of the libraries. The numbers of differentially expressed miRNAs among the three libraries and the numbers of their predicted targets are shown in Table 2. The GO analysis and KEGG pathway enrichment of most of the target genes of the conserved miRNAs are listed in Table 3. Intriguingly, miR400 was predicted to target genes that encode two MYB-like proteins that might be involved in the early developmental stage of *P*. *balfouriana* tissues. Several miRNAs (miR156, 166, and 172) were more abundant in the 3.6 μM library, which had a higher maturation rate, than in the other two libraries. Both miR1315 and miR5638, which targeted a gene encoding a receptor-like protein kinase, were upregulated in the 2.5 μM and 5.0 μM treatments compared to the 3.6 μM treatment. MiR164, which was predicted to target a gene encoding the AP2 domain-containing transcription factor, was downregulated in the 3.6 μM library.

**Table 2:** Numbers of differentially expressed miRNAs and their targets among the three 6-BAP-treated libraries.

	2.5 μM vs 5.0 μM	3.6 μM vs 2.5 μM	3.6 μM vs 5.0 μM
**miRNA**	79	91	87
**Target gene**	689	462	547

**Table 3:** GO analysis and KEGG pathway enrichment of target genes of known miRNAs.

	First^a^	Second^b^	Third^c^
**Cellular component**^d^	cell	intracellular	organelle
**Molecular function**^d^	binding	catalytic activity	hydrolase activity
**Biological process**^d^	cellular process	metabolic process	cellular metabolic process
**Pathway enrichment**^e^	metabolic pathways	spliceosome	RNA transport

^a, b, c^First, second, and third most abundant terms.

^d^GO categories.

^e^Kegg pathways.

## Prediction of potentially novel *P*. *balfouriana* miRNAs

In addition to the conserved miRNAs, 70, 54, and 57 potentially novel *P*. *balfouriana* miRNAs were predicted from the remaining unique unannotated sRNA sequences in the 2.5 μM, 3.6 μM, and 5.0 μM libraries, respectively (Table 4). The lengths of the putative novel miRNAs varied from 19 nt to 25 nt, of which 51.61% were 21 nt long. Based on the secondary structures predicted using Mfold, the precursor sequences had negative folding free energies ranging from -18.00 kcal mol^−1^ to -160.16 kcal mol^−1^ with an average free energy of -44.22 kcal mol^−1^, which is lower than the folding free energies reported for Arabidopsis (-59.5 kcal mol^−1^) and rice (-71.0 kcal mol^−1^) precursor miRNAs [47], but higher than for *Dimocarpus longan* Lour. (44.01 kcal mol^−1^) precursor miRNAs [24]. The numbers of reads obtained for those candidate novel miRNAs of *P*. *balfouriana* varied from 5 to 13,021. For example, ptc-miR167f (44 reads), mdm-miR391 (101 reads), and pta-miR946a (605 reads) were highly expressed, while zma-miR169r (6 reads), ath-miR160a (8 reads), and ath-miR169b (11 reads) were expressed at low levels. When nucleotide bias was analyzed, the nucleotide U (53.08%) was most frequent, followed by G (27.53%), A (16.01%), and C (3.38%).

**Table 4:** Predicted novel miRNAs of *Picea balfouriana*.

Treatment	Types of predicted miRNAs	Number of miRNAs	Predicted miRNAs	Target sites	Length (nt)	miRNA sequences matched to miRbase^a^
**2.5 μM**	94	86,595	70	180	21	18 (6, 33.3%)
**3.6 μM**	78	38,408	54	144	21	15 (8, 53.3%)
**5.0 μM**	77	50,498	57	152	21	13 (7, 53.8%)

^a^. The superscript letter a indicates that the novel miRNA sequence had orthologs in other species

To determine whether the novel miRNAs were conserved in other plants, we compared their sequences with the miRNA sequences of other organisms present in miRBase 21. The nine miRNAs listed in Table 5 had orthologs in other species.

**Table 5:** Orthologs of putative novel miRNAs conserved in other species.

miRNA	Location^a^	Sequence (5′–3′)	Length (nt)	Count	Homolog	MFE^b^ (kcal mol^−1^)	5′/3′
***pba-miR1***	**Spruce91_Unigene_BMK.6580:145:356:+**	CAGCCCTTCTGCTATCCACAAC	22	605	pta-miR946a	−76.7	3
***pba-miR2***	**Spruce93_Unigene_BMK.18933:170:269:-**	TGCCTGGCTCCCTGTATGCCA	21	8	ath-miR160a	−47.4	5
***pba-miR3***	**Spruce93_Unigene_BMK.27721:104:213:+**	TGAAGCTGCCAGCATGATCTGG	22	39	ath-miR167d	−56.2	5
***pba-miR4***	**Spruce93_Unigene_BMK.27721:101:215:+**	AGATCATGCGGCAGTTTCACC	21	44	ptc-miR167f	−59.6	3
***pba-miR5***	**Spruce93_Unigene_BMK.25825:32:161:-**	GGCAAGTTGTCTTTAGCTACA	21	6	zma-miR169r	−53.2	3
***pba-miR6***	**Unigene12051_C1907:73:212:+**	CAGCCAAGGATGACTTGCCGG	21	11	ath-miR169b	−51.5	5
***pba-miR7***	**Unigene29362_C1907:111:204:+**	CGCTATCCATCCTGGGCTTCA	21	22	aly-miR390a	−50.1	3
***pba-miR8***	**Unigene1519_C1907:71:158:+**	TCGCAGGATAGATGGCGCCGGCC	23	129	mdm-miR391	−46.1	5
***pba-miR9***	**Unigene42283_C1907:36:134:+**	TATGGGAGGAATGGGCAAAGCT	22	18	gma-miR482b	−41.1	3

^a^. The superscript letter a indicates the location of the miRNA on their precursors.

^b^. The superscript letter b means minimum free energy.

## Putative functions of predicted miRNA targets in *P*. *balfouriana*

GO terms for biological process, molecular function, and cellular component categories were assigned as targets of the novel miRNAs by BLAST searches against the *P*. *balfouriana* transcriptome databases. Under biological process, the predominant terms were primary metabolic process, metabolic process, cellular process, and other developmental processes. Under molecular function, the predominant terms were transferase activity, nucleic acid binding, binding, and other functions. Under cellular component, the predominant terms included cell, organelle, intracellular, and other components. Most of the predicted novel miRNA target genes were annotated as function unknown. The KEGG pathway enrichment showed that these target genes were involved in ribosome, metabolic pathways, glutathione metabolism, calcium signaling pathway, and other pathways.

### Validation of suitable reference genes for studying miRNA expression

#### geNorm

For each tissue, the gene-stability value (M) was calculated by geNorm for each candidate gene based on non-normalized expression levels (Q). The candidate genes were ranked according to the M value. An M value of 1.5 was used as a cutoff to assess gene stability [51–53]. For the 2.5 μM, 3.6 μM, and 5.0 μM sample groups, all the candidates had an M value lower than 0.8. The candidate reference genes csi-snoR14 and 5.8S rRNA were the least stable across the three samples (Table 6).

**Table 6:** Expression stability and ranking of reference genes as calculated by geNorm.

Samples	U6 snRNA	dlo-miR24	dlo-miR168a*	csi-snoR14	5.8S rRNA
**2.5 μM**	0.0079	0.0232	0.0001	0.0016	1.0000
**3.6 μM**	0.0041	0.0283	0.0001	0.0018	0.7013
**5.0 μM**	0.0056	0.0306	0.0000	0.0014	0.9188
**M < 1.5**	0.5900	0.5370	0.7350	0.4480	0.4750

#### BestKeeper

The main parameters used to evaluate a potential reference gene in BestKeeper are “std dev [± CP]” (recommend < 1) or “set dev [± x-fold]” (recommend < 2). These two parameters were the smallest for csi-snoR14 and dlo-miR24, indicating that they were the two most stable reference genes for the 2.5 μM, 3.6 μM, and 5.0 μM samples (Table 7).

**Table 7:** Stability assessment of the candidate reference genes by BestKeeper.

	U6 snRNA	dlo-miR24	dlo-miR168a*	csi-snoR14	5.8Sr RNA
**n**	9	9	9	9	9
**geo Mean [CP]**	30.46	28.19	36.76	32.29	23.20
**ar Mean [CP]**	30.46	28.19	36.77	32.29	23.20
**min [CP]**	29.84	27.80	35.29	31.89	22.86
**max [CP]**	31.88	28.69	37.81	32.66	24.10
**std dev [± CP]**	0.46	0.28	0.58	0.19	0.30
**CV [% CP]**	1.50	0.98	1.58	0.59	1.31
**min [x-fold]**	-1.54	-1.31	-2.78	-1.32	-1.27
**max [x-fold]**	2.69	1.41	2.06	1.29	1.87
**std dev [± x-fold]**	1.37	1.21	1.49	1.14	1.23

#### NormFinder

Expression stability of the candidate reference genes was reanalyzed with NormFinder. Expression variation of the candidate genes among the 2.5 μM, 3.6 μM, and 5.0 μM samples was estimated using a model-based approach. Intragroup variation was calculated and converted into a stability value for each candidate, and the candidates were ranked accordingly (Table 8). Among the five candidate reference genes, csi-snoR14 was the most stable with a value of 0.1167, and dlo-miR168a* was the least stable (0.4729).

**Table 8:** Stability assessment of the candidate reference genes by NormFinder.

Gene name	Stability value
**U6 snRNA**	0.3409
**dlo-miR24**	0.2486
dlo-miR168a*	0.4729
**csi-snoR14**	0.1167
**5.8S rRNA**	0.1812

### Validation of miRNAs and their potential targets

**Fig 4:**
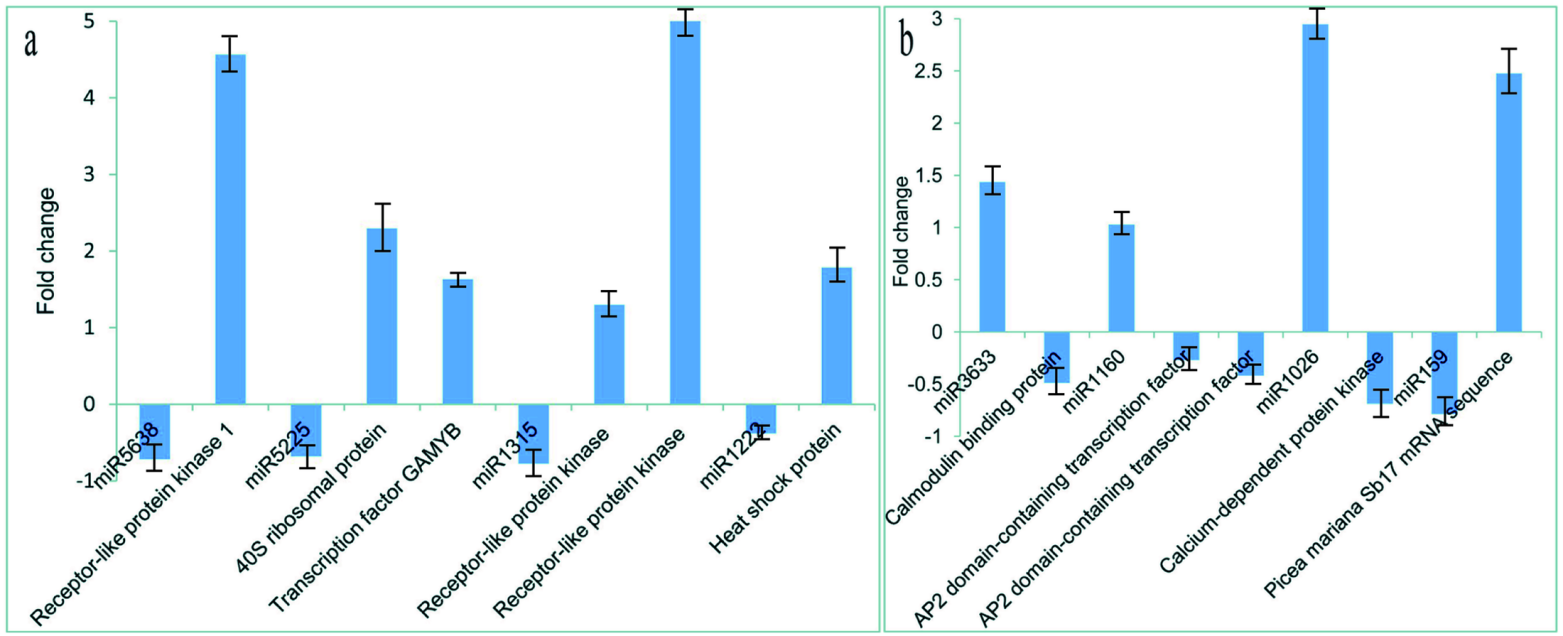
Validation of differentially regulated miRNAs and their targets by qRT-PCR. (a)The x-axis shows the miRNAs validated in this study. The y-axis shows the log_2_ ratio of their expression in the 3.6 μM 6-BAP versus the 5.0 μM 6-BAP libraries. Three biologically independent replicates were analyzed for each qRT-PCR; **(b)**The x-axis shows the miRNAs validated in this study. The y-axis shows the log_2_ ratio of their expression in the 3.6 μM 6-BAP versus the 2.5 μM 6-BAP libraries. Three biologically independent replicates were analyzed for each qRT-PCR.

The analyses of the five candidate reference genes identified csi-snoR14 as the optimal reference miRNA, and therefore all the miRNAs were normalized to csi-snoR14. The validation results showed that some miRNAs were upregulated in the 3.6 μM sample, which had the highest embryogenic competence, while others were downregulated. Eleven mRNAs were predicted as potential targets for eight miRNAs; both miR5225 and miR1160 had two potential targets. A gene encoding a receptor-like protein kinase was the predicted target of miR5638 and miR1315. Our results showed that five targets (receptor-like protein kinase, 40S ribosomal protein, transcription factor GAMYB, and heat shock protein) were clearly less abundant in the tissue with the lowest embryogenic ability (5.0 μM 6-BAP) compared with the tissue with the highest embryogenic ability (3.6 μM 6-BAP) (Fig 4a), while the abundances of the corresponding miRNAs (miR5638,-5225, -159, -1315, and -1222) were the opposite. Furthermore, miR3633, -1160, and -1026 were downregulated in tissues treated with 3.6 μM 6-BAP compared with those treated with 2.5 μM 6-BAP (Fig 4b), while the expression of their targets, AP2, calmodulin-binding protein, and calcium-dependent protein kinase, respectively, showed the opposite pattern. Finally, of seven potential novel miRNAs that were present in at least two of the 6-BAP treatments, six had homologous sequences in other species; the exception was spruce91-m0017, for which no homologous sequence was found, although a homologous sequence (lysine-rich arabinogalactan protein) was found for its putative target (Unigene58367).

## Discussion

Our results indicated that 6-BAP affected the production of somatic embryos and their germination rates. Moreover, 6-BAP influenced plant hormone levels and antioxidant enzyme activities in both the proliferation and maturation stages, showing that early embryo differentiation without plant growth regulators did not eliminate the influence of 6-BAP on the plant hormone levels and antioxidant enzyme activities in callus tissue.

Three receptor-like protein kinases were upregulated in the 3.6 μM 6-BAP treatment. Kinases such as SOMATIC EMBRYO RECEPTOR KINASE (SERK) have been identified previously in SE [54]. These kinases constitute a special subgroup of receptor protein kinases that are associated with SE. SERK1 is highly expressed during embryogenic cell formation in culture and during early embryogenesis in Arabidopsis. Hecht et al. [55] showed that overexpression of SERK1 not only did not result in any obvious plant phenotypes, it also gave a 3- to 4-fold increase in embryogenic competence, indicating that SERK1 enhanced embryogenic competence and promoted the transition of somatic cells to an embryogenic state. Thus, higher embryogenic ability may result from increased expression of receptor-like protein kinases by 6-BAP through miR5638 and miR1315. Previous studies have reported the effect of 6-BAP on *SERK* genes. For example, 6-BAP alone induced SE in *Medicago truncatula* Gaertn. and promoted *MtSERK*1 expression [56]. Nolan et al. [56] reported that auxin and naphthalene-1-acetic acid application together with 6-BAP could significantly stimulate embryogenic cell formation and proliferation, which was accompanied by increased *MtSERK1* expression. However, our results differ from those obtained in *M*. *truncatula*, but are consistent with those of Zhang et al. [57], who demonstrated that 6-BAP inhibited SE and reduced ZmSERK1 and ZmSERK2 expression in a maize culture.

Notably, a gene targeted by miR5225 that encodes the transcription factor GAMYB was upregulated in the 3.6 μM 6-BAP treatment. GAMYB is involved in programmed cell death in both aleurone and tapetal tissues, and in both tissues this process is mediated by a gibberellin (GA_3_) [58]. Conversely, members of the miR159 family repressed conserved GAMYB-like genes [59–61]. Noma et al. [62] found lower GA (probably GA_1_) levels in embryogenic lines of carrot and anise, but Jiménez and Bangerth [63] found higher GA (GA_1_, GA_3_, GA_20_) levels in embryogenic maize lines. Furthermore, in carrot [64], wheat [65], and grapevine [66], no differences in GA levels were found among cultures showing different embryogenic characteristics. A few researchers have studied the relationships among GAs and cytokinins. We found that the GA_3_ content in the tissues with highest embryogenic ability (treated with 3.6 μM 6-BAP) was higher than in those treated with 5.0 μM 6-BAP. On the other hand, GAMYB has been identified as an activator of GA-regulated genes [59]. Together, these findings indicate that miR159 may repress GAMYB expression in the 5.0 μM treatment, leading to reduced GA_3_.

Some members of the AP2 domain-containing transcription factor family, which contains 173 members [67], were reported to play a major role in embryogenesis and organ development [68,69]; for example, BABY BOOM (BBM) was shown to be involved in cell proliferation and morphogenesis [70,71]. The embryogenic tissue of Arabidopsis, which can induce SE, had elevated BBM expression [72]. Piyatrakul et al. [73] identified 11 AP2/ERF genes as very early markers that could predict the regeneration potential of proliferating callus lines. However, how these genes regulated early SE was unclear. Furthermore, 12 miRNAs (miR156, -159, -172, -393, -395, -396, -408, -894, -1511, -n11, -n12, and -n14) were predicted to inhibit the transcripts of 29 *Hevea brasiliensis* (Willd. ex A. Juss.) Müll. Arg. HbAP2/ERF genes [67]. In our study, we identified two AP2 domain-containing transcription factors that may be regulated by miR1160, which were downregulated in the tissues with higher embryogenic ability, indicating that the overexpression of AP2 triggered by miR1160 may lead to a decrease or even a loss of embryogenic competence.

Calmodulin-binding protein and calcium-dependent protein kinase (CDPK) were downregulated in the 3.6 μM treatment compared to the 2.5 μM treatment. In carrot, the role of calcium in SE is essential for morphogenesis of undifferentiated cells into somatic embryos at a threshold of 200 mM [74]. Higher concentrations of calcium have no effect on either the viability or embryogenic potential of the culture. At lower concentrations, or after chelation of residual calcium with ethylene glycol-bis(2-aminoethylether)-*N*,*N*,*N′*,*N′*-tetraacetic acid, SE is inhibited and the calcium channel blockers, verapamil and nifedipine, exert an inhibitory influence on embryogenic capacity [75]. Two CDPKs of 55 and 60 kDa have been identified in soluble protein extracts of sandalwood embryogenic cultures; these have Ca^2+^-dependent and calmodulin-independent protein kinase activity and a developmentally regulated, tissue-specific soluble CDPK (swCDPK) accumulates in all stages of embryo development [76,77]. This indicates that swCDPK is a Ca^2+^ modulator that can act alone or in conjunction with calmodulin during sandalwood SE.

## Conclusions

In the present study, sRNA libraries were constructed by high-throughput sequencing of *P*. *balfouriana* callus cultures treated with three 6-BAP concentrations. After processing the sequencing data, we identified more than 4,000 conserved and 50 novel miRNAs in each library. The expression levels of more than 70 miRNAs showed significantly different regulation between pairs of treatments. The expression patterns for eight selected miRNAs and their targets were examined in detail, and there was a negative correlation between the expression patterns of the miRNAs and their targets. Notably, these targets have been reported to be involved in SE, suggesting that the associated miRNAs might act as regulators of embryogenic ability. The characterization and expression profile comparisons of the *P*. *balfouriana* miRNA libraries provide a good foundation for elucidating the complex miRNA-mediated regulatory network of SE in callus tissue treated with 6-BAP.

## Supporting information

S1 Table Selected miRNAs and primer sequences. (DOC)

S2 Table The 30 target mRNAs and their primer sequences. (DOCX)
