# Supplementary material for: Identification of novel miRNAs and miRNA expression profiling in embryogenic tissues of Picea balfouriana treated by 6-benzylaminopurine
Source: PLoS One. 2017 May 9;12(5):e0176112. doi: 10.1371/journal.pone.0176112 (PMC5423612; doi:10.1371/journal.pone.0176112)
Supplement: S1 Table — (DOC) [file pone.0176112.s001.doc]

| miRNA | F | R |
| --- | --- | --- |
| miR1026 | GAGAAAGACTGGAAGAGG | GTCGTATCCAGTGCGTGTCGTGGAGTCGGCAATTGCACTGGATACGACTGCCTC |
| miR1160 | TGACAAGGAAGAGGAGG | GTCGTATCCAGTGCGTGTCGTGGAGTCGGCAATTGCACTGGATACGACGTCCTC |
| miR1222 | TTAAGGAGTTGTTGGATA | GTCGTATCCAGTGCGTGTCGTGGAGTCGGCAATTGCACTGGATACGACTATCCA |
| miR1315 | GGAGGCTCGTCAGGTTCC | GTCGTATCCAGTGCGTGTCGTGGAGTCGGCAATTGCACTGGATACGACTTGGGA |
| miR159 | CTTGGATTGAAGGGAGCT | GTCGTATCCAGTGCGTGTCGTGGAGTCGGCAATTGCACTGGATACGACGGAGCT |
| miR3633 | TGAATGATGGTTTGGAGG | GTCGTATCCAGTGCGTGTCGTGGAGTCGGCAATTGCACTGGATACGACCCTCCA |
| miR5225 | CTGTGTAGGAGAGATGC | GTCGTATCCAGTGCGTGTCGTGGAGTCGGCAATTGCACTGGATACGACCGCATC |
| miR5638 | ACAGTGGTCAGGTGGGT | GTCGTATCCAGTGCGTGTCGTGGAGTCGGCAATTGCACTGGATACGACAACCCA |
| Spruce91-m0009 | CAGCCCTTCTGCTATCCA | GTCGTATCCAGTGCGTGTCGTGGAGTCGGCAATTGCACTGGATACGACGTTGTG |
| Spruce93-m0032 | AGATCATGCGGCAGTTTC | GTCGTATCCAGTGCGTGTCGTGGAGTCGGCAATTGCACTGGATACGACGGTGAA |
| Spruce93-m0055 | CGCTATCCATCCTGGGCT | GTCGTATCCAGTGCGTGTCGTGGAGTCGGCAATTGCACTGGATACGACTGAAGC |
| Spruce93-m0060 | TATGGGAGGAATGGGCAA | GTCGTATCCAGTGCGTGTCGTGGAGTCGGCAATTGCACTGGATACGACAGCTTT |
| Spruce93-m0044 | TCGCAGGATAGATGGCGC | GTCGTATCCAGTGCGTGTCGTGGAGTCGGCAATTGCACTGGATACGACGGCCGG |
| Spruce93-m0042 | CAGCCAAGGATGACTTGC | GTCGTATCCAGTGCGTGTCGTGGAGTCGGCAATTGCACTGGATACGACCCGGCA |
| Spruce91-m0017 | AGAGACAAAACAACAGGAG | GTCGTATCCAGTGCGTGTCGTGGAGTCGGCAATTGCACTGGATACGACATCTCC |
| dlo-miR24 | AAATGATTTCGGACCAGG | GTCGTATCCAGTGCGTGTCGTGGAGTCGGCAATTGCACTGGATACGACAGCCTG |
| dlo-miR168* | ACGCCTTGCATCAAAGGG | GTCGTATCCAGTGCGTGTCGTGGAGTCGGCAATTGCACTGGATACGACATGCGA |
| Csi‐snoR14 | CATGTCTGTCAATCCACTG | GTCGTATCCAGTGCGTGTCGTGGAGTCGGCAATTGCACTGGATACGACAACCTG |
| 5.8S rRNA | AGTGTGAATTGCAGAATCC | GTCGTATCCAGTGCGTGTCGTGGAGTCGGCAATTGCACTGGATACGACCGACCG |
| U6 snRNA | CGATACAGAGAAGATTAGC | GTCGTATCCAGTGCGTGTCGTGGAGTCGGCAATTGCACTGGATACGACCCATGC |
