# Supplementary material for: Identification of novel miRNAs and miRNA expression profiling in embryogenic tissues of Picea balfouriana treated by 6-benzylaminopurine
Source: PLoS One. 2017 May 9;12(5):e0176112. doi: 10.1371/journal.pone.0176112 (PMC5423612; doi:10.1371/journal.pone.0176112)
Supplement: S2 Table — (DOCX) [file pone.0176112.s002.docx]

| Target mRNA | F | R |
| --- | --- | --- |
| Spruce91_Unigene_BMK.14625 | GGTGCAGTCTATAATCTCAAAACGAC | CAAGACACATCCTCTCTGCGG |
| Spruce91_Unigene_BMK.708 | AAGTCTCGGGGCGGTTTAGT | TATAAGAGCTGTGACGAAC |
| Spruce91_Unigene_BMK.8428 | AAAGGTCACACTGGGCAGGG | GGACAAAAAAGTATCCTCTTTTG |
| Spruce92_Unigene_BMK.11779 | ACACAAAACGATGAACACACCG | CGTTCTTAACTGTCTTCTCTCTCGTG |
| Spruce92_Unigene_BMK.13913 | TACCGAAGCGATAGACATCTGTG | ACTTTGTCGTTCGGTTCCTACTTTA |
| Spruce92_Unigene_BMK.228 | GAAGATCCGGGAAAAGGCC | CGTTTAACCGAACTTCCCTCG |
| Spruce93_Unigene_BMK.1346 | GGAGAGTAGTCAGTCGGCACAA | CGTTTCACACTTACTATCACACCTCAC |
| Spruce93_Unigene_BMK.14744 | GAAAGACGCGAAAGAGAAGT | CTCCCGGTTCACCTTCTTC |
| Spruce93_Unigene_BMK.17039 | CAGAAGAGGCGGGGTATAAG | CCTGTAACGTACAAAGAAG |
| Spruce93_Unigene_BMK.17415 | ACCTCGAGGGAAGTTAGGTT | CCTCTACATAACAGTGACC |
| Spruce93_Unigene_BMK.25825 | AAAAGGAGAATCGTCTAGTACTCACCTAC | CTTCGGTTCCTACTGAACGGTT |
| Spruce93_Unigene_BMK.28756 | GCATATAATGTGATGTCTTTA | CCTCGACCAAAGAAGATGCA |
| Spruce93_Unigene_BMK.40901 | CCTAGAGGTCAACGACGTCACA | GTATAAACGGTGTTCCTCCGTTTT |
| Spruce93_Unigene_BMK.6230 | ACCAGGTCTTTTAGGAGAAAG3 | TCATAATTCACGAGACTTTT |
| Unigene10412_C1907 | ACAACGACACGGACGACCA | TCACTACGTCAGACCACCCG |
| Unigene12940_C1907 | GGACCGCTTTACAGACAGGC | AGCCGCCCTGAGAGAGGT |
| Unigene18311_C1907 | AAATGCCTAGGATGCGCTCC3 | AGAGCCCTAATCGTCGCCTC |
| Unigene20257_C1907 | TGATAAAGAGGCCAAGCTAGGG | CCTCCGAGCAGTCCAACG |
| Unigene20258_C1907 | TGATAAAGAGGCCAAGCTAGGG | CCTCCGAGCAGTCCAACG |
| Unigene2484_C1907 | GACAACCCGCTGCTCTGG | CGTCTGTTGCTGTTACTCCTGCT |
| Unigene31249_C1907 | AAGTTACTCATCTGCTGTAGGTACCGA | CGTGCGGTCCTCTTTCTGAC |
| Unigene31442_C1907 | TTAGGGCAACTGAGGACGACA | TAAGTTCCTCCTTCTTCTCCTCCTT |
| Unigene31529_C1907 | AACTTCGTCTAGACAGACTG | GACTCCTTCATCTTCGAAAAG |
| Unigene58367_C1907 | GAATAGAGGCTACTACGGACGGG | CCTCTGCTTTGTTGTCTTCTGCT |
| Unigene60980_C1907 | AAGGCCTGTCCTTGTCCTC | ACGGACAGTCACTTGCTTAGA |
| Unigene6367_C1907 | TCGACGTTCTAGCATTCGGG | CCACCTATACTGTTTCTTCTCTTCCG |
| Unigene6501_C1907 | AGACGTCTCCTTCTTCATCCGA | AAGGTACTAGTTCTGTACCGACTCCTG |
| Unigene7974_C1907 | ATTCTGATTCTGAGGACCTGGG | TCGTTCTCGTGCTTCTCGTCTA |
| Unigene7978_C1907 | ATTCTGATTCTGAGGACCTGGG | TCGTTCTCGTGCTTCTCGTCTA |
| Unigene8360_C1907 | CGAAGTCGTTGGCGGACTT | GTCCAACGTACTTTCTCAACAACCT |
